# Electrical Manipulation of Magnetic Domain Structure in van der Waals Ferromagnetic Fe_3_GaTe_2_ Using Ferroelectric PMN‐PT Single Crystal

**DOI:** 10.1002/advs.202503530

**Published:** 2025-08-27

**Authors:** Riku Iimori, Yuta Kodani, Shaojie Hu, Takashi Kimura

**Affiliations:** ^1^ Department of Physics Kyushu University 744 Motooka Fukuoka 819‐0395 Japan; ^2^ College of Integrated Circuits and Optoelectronic Chips Shenzhen Technology University 3002 Lantian Road, Pingshan District Shenzhen Guangdong 518118 China; ^3^ Recerch Center for Semiconductor and Device Ecosystem Kyushu University 6‐1 Kasugakoen Kasuga 816‐8580 Japan

**Keywords:** Dzyaloshinskii–Moriya interaction, magnetic anisotropy, multiferroic, van‐der Waals ferromagnet

## Abstract

2D van der Waals (vdW) ferromagnets have emerged as promising materials for spintronic applications due to their unique magnetic properties and tunability. Controlling ferromagnetism via external stimuli is critical for both fundamental research and device integration. In particular, modulation of magnetic anisotropy and exchange interactions through strain offers a viable pathway for functional control. Owing to their weak interlayer coupling, vdW ferromagnets exhibit pronounced sensitivity to strain, enabling effective tuning of their magnetic states. In this study, electric‐field‐induced magnetoelectric coupling is investigated in the above‐room‐temperature vdW ferromagnet Fe_3_GaTe_2_ integrated on a ferroelectric PMN‐PT substrate. It is demonstrated that application of an electric field leads to a substantial reduction in coercive force along with dynamic reconfiguration of the magnetic domain structure. These effects are attributed to electric‐field‐induced modulation of the vdW interlayer gap and enhancement of the Dzyaloshinskii–Moriya interaction. These findings reveal a strong interplay between electric fields and magnetism in vdW systems, offering a viable route toward the development of low‐power, multifunctional magnetic devices. This work establishes a foundation for the electric‐field control of magnetic properties in vdW ferromagnets and highlights their potential in next‐generation spintronic technologies.

## Graphical abstract

2D van der Waals ferromagnets enable tunable magnetism for spintronics. This study demonstrates electric‐field‐induced control of Fe_3_GaTe_2_ magnetism on a PMN‐PT substrate, reducing coercive force and altering domain structures. The effects stem from interlayer gap modulation and enhanced DzyaloshinskiiMoriya interaction, establishing a path toward low‐power, multifunctional magnetic devices through electric‐field control of vdW ferromagnets.

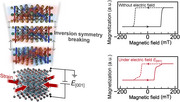

## 1 Introduction

2D van der Waals (vdW) ferromagnets have garnered significant attention due to their potential to host novel magnetic phenomena and their applicability in low‐dimensional spintronic systems.^[^ ^1^, ^2^, ^3^, ^4^, ^5^, ^6^, ^7^, ^8^ ^]^ The distinctive crystal structure of these materials, composed of atomic layers with chemically inert surfaces held together by weak vdW interactions, provides an unparalleled platform for exploring the interplay between 2D material properties and magnetism.^[^ ^1^, ^9^, ^10^, ^11^, ^12^, ^13^ ^]^ This structural configuration enables the coexistence of low dimensionality and magnetic order, leading to intriguing phenomena such as flat‐band magnetism,^[^ ^14^, ^15^ ^]^ the tunable quantum anomalous Hall effect,^[^ ^16^, ^17^, ^18^, ^19^, ^20^ ^]^ and room‐temperature magnetic skyrmions.^[^ ^21^, ^22^, ^23^, ^24^, ^25^ ^]^ From a technological perspective, vdW ferromagnets are particularly attractive because of their compatibility with the fabrication of atomic‐layer devices via mechanical exfoliation. Their ultra‐thin and mechanically robust nature makes them ideal candidates for flexible and stretchable electronic components, including wearable devices.^[^ ^26^, ^27^ ^]^ These properties also position vdW ferromagnets as promising materials for micro‐electro‐mechanical systems (MEMS) sensors, enabling novel operational mechanisms.

Despite these advantages, the stability of ferromagnetic order in 2D vdW materials remains a significant challenge. Although some materials exhibit Curie temperatures (*T*_C_) near room temperature,^[^ ^28^, ^29^ ^]^ the inherently low magnetic moments and susceptibility to thermal fluctuations in 2D systems hinder their practical applicability. As predicted by the Mermin‐Wagner theorem, isotropic 2D systems with short‐range interactions cannot sustain long‐range magnetic order.^[^ ^30^ ^]^ However, magnetic anisotropy can stabilize such order, making it a critical parameter in defining the ferromagnetic properties of 2D materials. Addressing these challenges requires the discovery and development of vdW materials with enhanced magnetic anisotropy and stronger ferromagnetic interactions. One promising candidate is Fe_3_GaTe_2_, a vdW ferromagnet that exhibits ferromagnetic order above room temperature (*T*_C_ ∼ 365 K) with pronounced perpendicular magnetic anisotropy.^[^ ^31^ ^]^ Additionally, Fe_3_GaTe_2_ demonstrates high electrical and thermal spin‐conversion efficiencies,^[^ ^32^ ^]^ positioning it as a key material for next‐generation spintronic devices. Building on these findings, we investigated the impact of applied pressure on the magnetic properties of Fe_3_GaTe_2_ thin films.^[^ ^33^ ^]^ Our study revealed that pressure systematically reduces the vdW interlayer gap, thereby enhancing perpendicular magnetic anisotropy at room temperature under relatively low pressures (below 1 GPa). Such behavior is rarely observed in conventional three‐dimensional magnetic materials. These results underscore a strong coupling between the vdW gap and magnetic anisotropy, highlighting the exceptional pressure responsiveness of Fe_3_GaTe_2_. This discovery provides critical insights into the tunability of magnetic properties in 2D vdW ferromagnets, paving the way for innovative applications in spintronics and flexible electronics.

Expanding upon the idea of pressure‐modulated magnetism in Fe_3_GaTe_2_, we explored the potential for significant modulation of magnetic properties induced by in‐plane strain transmitted from a substrate. Specifically, we investigated the use of ferroelectric substrates, where strain can be dynamically controlled through the application of an electric field. Previous studies have extensively analyzed field‐induced modulation of magnetic properties in magnetic‐ferroelectric hybrid devices.^[^ ^34^, ^35^, ^36^, ^37^, ^38^, ^39^ ^]^ The ferroelectric piezoelectric effect enables strain‐mediated magnetic modulation solely via electric fields, eliminating current losses and offering a promising approach for nonvolatile memory technologies based on novel operating principles. Moreover, the anisotropic piezoelectric strain and strain gradients generated by ferroelectric substrates can introduce additional symmetry breaking in vdW ferromagnets, potentially giving rise to emergent magnetic phenomena. To leverage these effects, we combined the ferroelectric piezoelectric properties of the substrate with the pronounced magnetoelastic coupling observed in Fe_3_GaTe_2_, targeting effective electric‐field modulation of its magnetic behavior.

**Figure 1:**
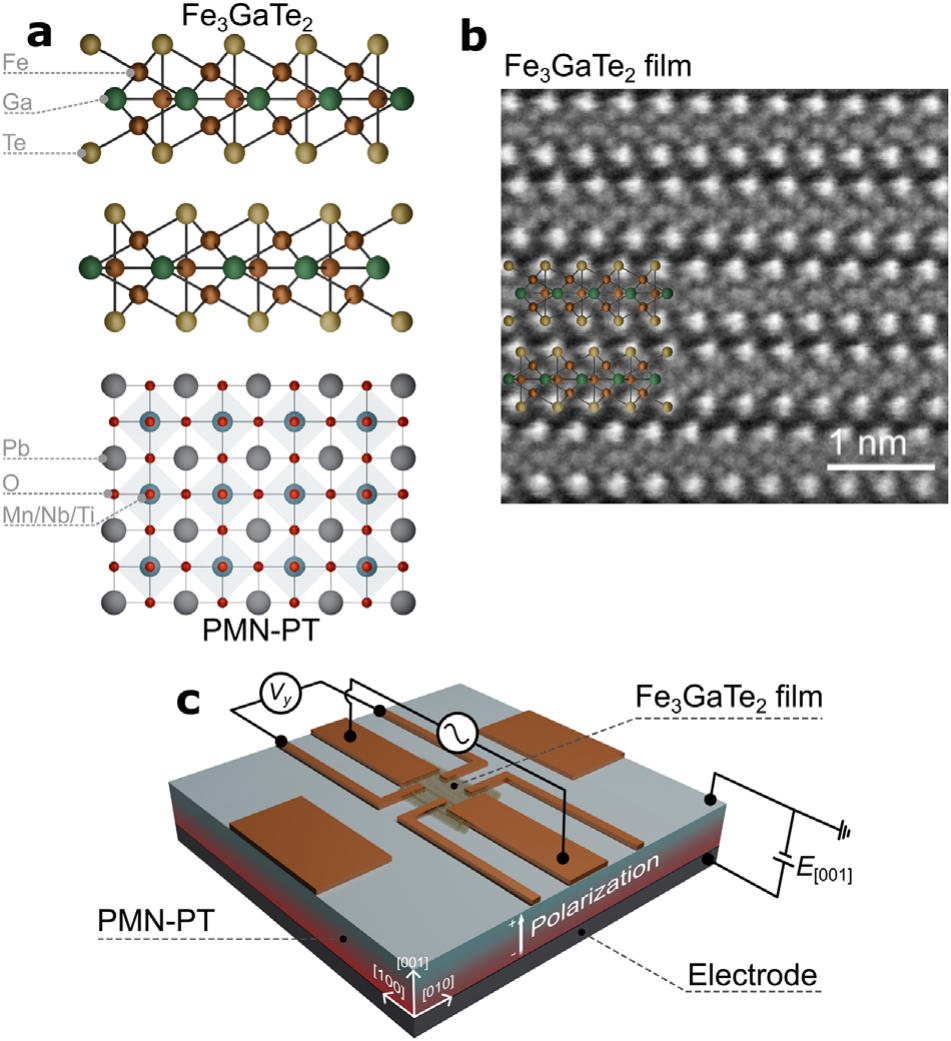
Heterostructure combining vdW ferromagnetic Fe_3_GaTe_2_ and ferroelectric PMN‐PT. a) Schematic of atomic structure of Fe_3_GaTe_2_/PMN‐PT heterostructure. Here, the composition of the PMN‐PT is 0.7Pb(Mg_1/3_Nb_2/3_)O_3_‐0.3PbTiO_3_, and a *c*‐plane cut PMN‐PT substrate was used in this study. b) Cross‐sectional transmission electron microscopy (TEM) image of the Fe_3_GaTe_2_ film. c) Device structure for the magneto‐transport measurement with circuit diagram. The electric field is applied to the ferroelectric PMN‐PT substrate in a back‐gate configuration. The inverse piezoelectric effect of the substrate transmits the strain to the Fe_3_GaTe_2_.

In this study, we employed the ferroelectric crystal 0.7Pb(Mg_1/3_Nb_2/3_)O_3_‐0.3PbTiO_3_ (PMN‐PT), selected for its excellent ferroelectric characteristics, including a Curie temperature (*T*_C_) of approximately 416 K, a high piezoelectric charge coefficient (*d*_33_ ∼ 2000 pC/N), and an electromechanical coupling factor (*k*_33_ ∼ 0.94).^[^ ^40^, ^41^ ^]^ As illustrated in **Figure** **1**a, we integrated PMN‐PT with Fe_3_GaTe_2_ to examine electric‐field‐induced effects on the magnetic properties of the vdW ferromagnet. Our experimental results demonstrate that the magnetic domain structure in Fe_3_GaTe_2_ can be effectively manipulated at room temperature through the inverse piezoelectric effect of PMN‐PT under an applied electric field. These findings provide critical insights into the magnetic behaviors at vdW ferromagnet/ferroelectric interfaces and underscore the potential for energy‐efficient electric‐field control of magnetic structures. This work highlights a pathway for developing advanced spintronic devices with reduced power consumption and enhanced functional tunability.

## 2 Results and Discussion

To investigate the magnetic properties of the Fe_3_GaTe_2_/PMN‐PT heterostructure, we performed anomalous Hall effect (AHE) measurements. Figure 3c shows the transverse resistance (*R*_*xy*_) curves observed at various temperatures. For ferromagnetic materials, the Hall resistance can be expressed as:^[^ ^42^, ^43^, ^44^ ^]^

(1) $$R_{xy}=R_{\mathrm{OHE}}B_{z}+R_{\mathrm{AHE}}M_{z},$$

where the first term represents the ordinary Hall effect (OHE), and the second term corresponds to the anomalous Hall effect (AHE). The OHE contribution was neglected in this study due to its negligible magnitude compared to the dominant AHE component. The *R*_*xy*_ curve at room temperature (*T* = 297 K) exhibits a nearly perfect rectangular hysteresis loop, indicative of robust perpendicular magnetic anisotropy under the standard conditions. Notably, the Fe_3_GaTe_2_ film prepared on a PMN‐PT substrate displayed a more pronounced fully magnetized state at zero magnetic field compared to a similar film deposited on a thermally oxidized silicon substrate (SiO_2_/Si). As the temperature increased beyond room temperature, the AHE curves revealed that the magnetization reversal process was accompanied by the formation of multi magnetic domains prior to achieving full saturation. Figure 3d illustrates the temperature dependence of the AHE resistance (Δ*R*_AHE_). Here, 2Δ*R*_AHE_ is defined as the difference in transverse resistance between magnetic fields μ_0_ *H* = +185 mT and μ_0_ *H* = −185 mT. Here, we performed a fitting of *R*_*xy*_ vs. *T* based on the relationship *R*_*xy*_∝(1 − *T*/*T*_C_)^β^. As a result, the Curie temperature *T*_C_ of the ferromagnetic phase was determined to be ∼338 K, which is consistent with the values reported in previous studies.^[^ ^31^, ^32^, ^33^ ^]^

**Figure 2:**
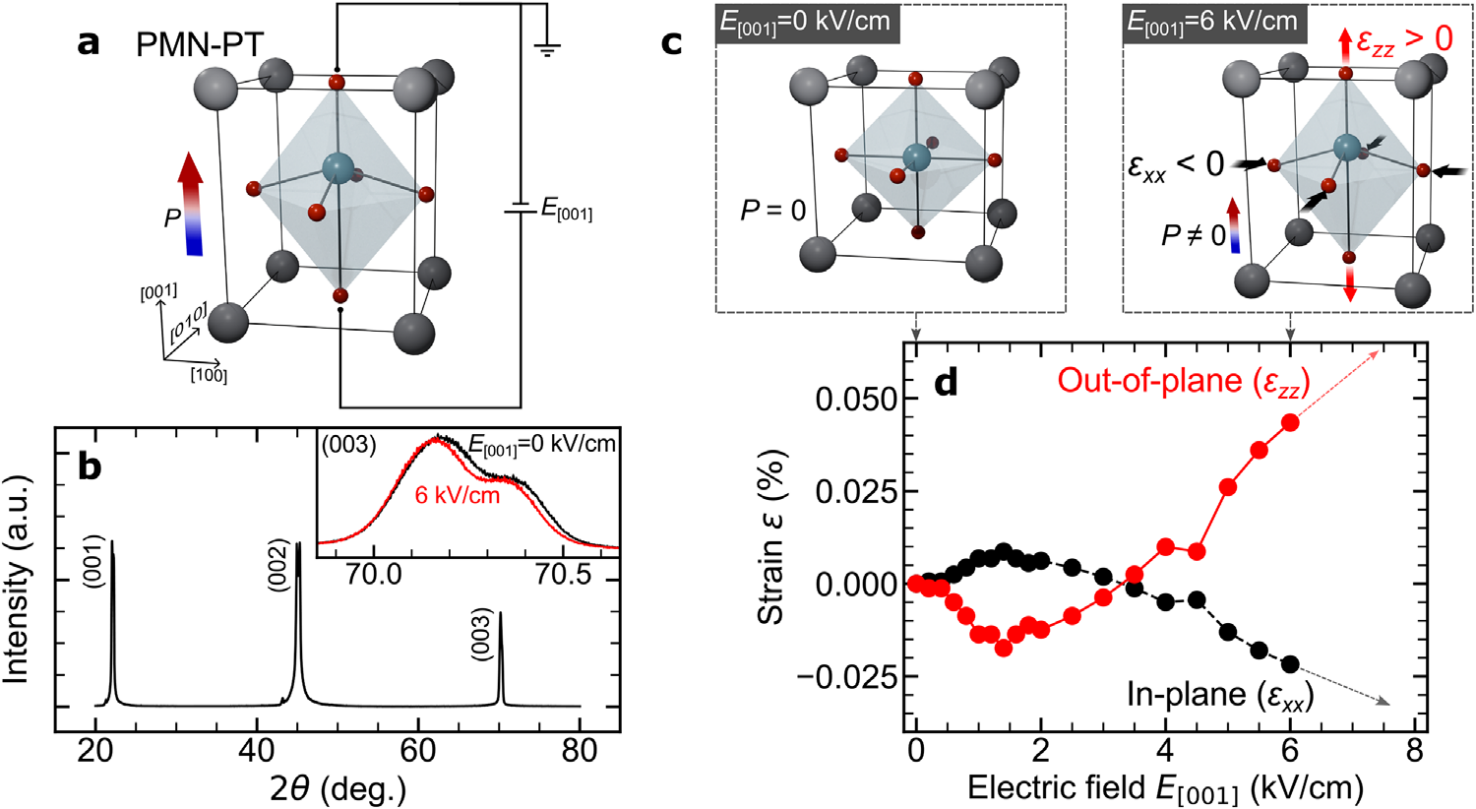
Electric field characteristics of the PMN‐PT single crystal. a) Schematic illustration of the inverse piezoelectric effect of the PMN‐PT crystal under the electric field *E*_[001]_. In this study, the electric field *E*_[001]_ is applied along the [001] direction of PMN‐PT single crystal. b) X‐ray diffraction (XRD) pattern of the PMN‐PT single crystal. The inset shows the (003) spectra as a function of the back‐gate electric field up to 6 kV/cm. c) Schematic diagrams of the polarization and strain profiles under *E*_[001]_ = 0 kV/cm and 6 kV/cm. d) Electric field dependence of out‐of‐plane strain (ε_*zz*_ = Δ*c*/*c*) evaluated from the shift of the (003) XRD peak. In‐plane (ε_*xx*_) strain was derived based on the approximation of constant volume in the Poisson effect (2ε_*xx*_ + ε_*zz*_ = 0).

We further examined the effect of an applied electric field on the AHE behavior of the Fe_3_GaTe_2_/PMN‐PT heterostructure. **Figure** **4**a,b presents the AHE curves for electric fields ranging from −12 kV/cm to +12 kV/cm. The results reveal that the rectangular hysteresis loop evolves into a magnetic hysteresis loop characterized by a multi‐domain structure under the application of an electric field (*E*_[001]_). This transformation was dependent on the magnitude of *E*_[001]_ but showed minimal sensitivity to the polarity of the spontaneous electric dipole in the PMN‐PT.

**Figure 3:**
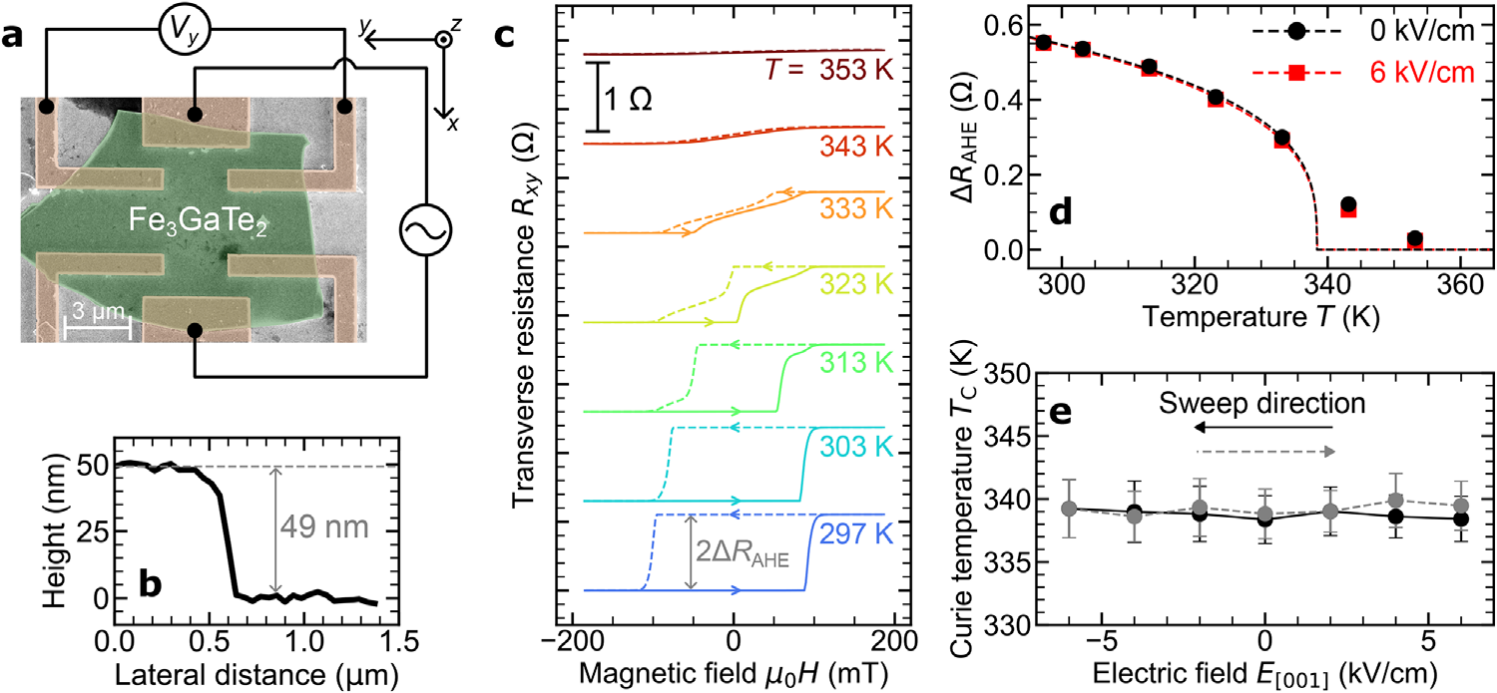
Ferromagnetic properties of the Fe_3_GaTe_2_/PMN‐PT device. a) Scanning electron microscope (SEM) image of the fabricated Fe_3_GaTe_2_/PMN‐PT device together with the probe configuration. b) Cross‐sectional height profile measured by the atomic force microscopy (AFM). c) Transverse resistance *R*_*xy*_ as a function of the applied magnetic field *H* along the perpendicular direction under various temperatures. d) Temperature dependence *T* of the anomalous Hall resistance Δ*R*_AHE_ at *E*_[001]_ = 0 and 6 kV/cm. Curie temperature *T*_C_ of the ferromagnetism for the Fe_3_GaTe_2_/PMN‐PT heterostructure was determined by fitting the Δ*R*_AHE_ vs. *T* data to the relationship Δ*R*_AHE_ = *A*(1 − *T*/*T*_C_)^β^. Here, *A*, *T*_C_ and β are fitting parameters. The fitting was conducted for the temperature range *T* ⩽ 333 K, which is below the Curie temperature. This range was chosen to avoid the influence of non‐monotonic changes in the anomalous Hall effect (AHE) near *T*_C_, which can arise from spin fluctuations and associated modifications in the electronic structure. For example, at an applied back‐gate electric field of *E*_[001]_ = 6 kV/cm, the fitting parameters were obtained as *A* = 1.06 ± 0.10 Ω, *T*_C_ = 338.4 ± 1.9 K, and β = 0.303 ± 0.044. e) Curie temperature *T*_C_ as a function of the applied electric field *E*_[001]_.

**Figure 4:**
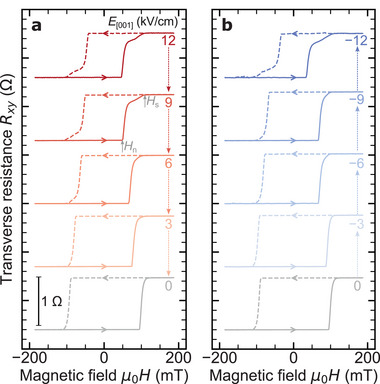
Electric‐field modulated anomalous Hall resistance curves in the Fe_3_GaTe_2_/PMN‐PT device. Room‐temperature anomalous Hall resistance *R*_*xy*_ curves measured under different back‐gate electric fields *E*_[001]_ ranging a) from 0 to +12 kV/cm (positive direction) and b) from 0 to −12 kV/cm (negative direction).

**Figure 5:**
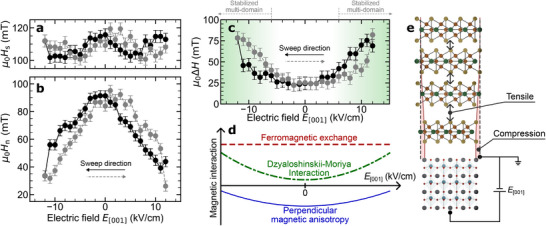
Origin of the electric field effect on the magnetic domains of the Fe_3_GaTe_2_/PMN‐PT heterostructure. a) Electric field *E*_[001]_ dependence of the saturation magnetic field *H*_s_. b) The nucleation magnetic field *H*_n_ as a function of the *E*_[001]_. Here, the black solid line represents the magnetic field being swept from positive to negative, while the gray dashed line indicates the opposite direction. c) Electric field *E*_[001]_ dependence of the Δ*H* (=*H*_s_ − *H*_n_) in the Fe_3_GaTe_2_/PMN‐PT heterostructure. d) Changes in magnetic interactions induced by electric field *E*_[001]_. Since there was no significant change in the Curie temperature, the ferromagnetic interactions remained largely unaffected. On the other hand, the in‐plane strain of PMN‐PT expands the van der Waals gap, leading to a reduction in perpendicular magnetic anisotropy. Additionally, the change in symmetry due to the strain gradient enhances the Dzyaloshinskii–Moriya interaction. e) Strain profile induced by the electric field in the Fe_3_GaTe_2_/PMN‐PT structure.

To further elucidate the magnetization process, we evaluated the domain nucleation field (*H*_n_) and the saturation field (*H*_s_), as shown in Figure 5a,b. As previously described, the application of an electric field transitions the magnetization process from an almost coherent rotation to a reversal mechanism associated with the formation of multi‐domains. Figure 5a,b presents the dependence of *H*_n_ and *H*_s_ on the electric field (*E*_[001]_), where *E*_[001]_ was initially swept from +12 kV/cm to −12 kV/cm and then returned to +12 kV/cm. *H*_n_ decreases monotonically with increasing electric field, exhibiting a reduction of approximately 70% under an applied field of 12 kV/cm. Notably, *H*_n_ displays a marked decrease with increasing magnitude of *E*_[001]_, forming a butterfly‐shaped profile consistent with strain curves observed in piezoelectric materials. This behavior suggests that the electric‐field effect on *H*_n_ in Fe_3_GaTe_2_ originates from strain induced by the inverse piezoelectric effect of the PMN‐PT substrate. Referring to the strain curve of the PMN‐PT substrate in **Figure** **2**d, compressive in‐plane strain is generated upon the application of *E*_[001]_. This strain is transferred to the Fe_3_GaTe_2_ layer, causing expansion along the *c*‐axis via the Poisson effect, particularly within the van der Waals (vdW) gap. Conversely, a slight increase in *H*_s_ was observed near zero electric field; however, this variation remained within the error margins. We also observed that the Curie temperature (*T*_C_) of Fe_3_GaTe_2_ is insensitive to electric fields (*E*_[001]_) up to 6 kV/cm, as presented in **Figure** **3**e. To avoid potential damage to the PMN‐PT substrate at elevated temperatures, the field dependence of *T*_C_ was not evaluated beyond 6 kV/cm. The absence of significant field dependence of *T*_C_ aligns with the minimal field sensitivity of *H*_s_.

To further investigate the origin of electric‐field effects on the magnetic properties of the Fe_3_GaTe_2_/PMN‐PT heterostructure, we analyzed the difference Δ*H* between the saturation field *H*_s_ and the nucleation field *H*_n_ of the multi‐domain structure. This difference characterizes the stabilization region of metastable domains. **Figure** **5**c illustrates the stabilization field range of multi‐domains as a function of the electric field. The results reveal that higher electric fields enhance the stability of the multi‐domain structure, indicating the pivotal role of electric‐field‐induced strain in modulating the magnetic properties of vdW ferromagnetic systems.

To understand the electric‐field dependence of Δ*H*, we simply propose that the observed reduction in perpendicular magnetic anisotropy arises from an expansion of the van der Waals (vdW) gap. Our previous studies on the pressure effects in Fe_3_GaTe_2_ have demonstrated that applying the pressure significantly enhances perpendicular magnetic anisotropy.^[^ ^33^ ^]^ According to the analysis, the pressure is known to induce the modification of the vdW gap as well as the lattice parameters effectively, namely a few percent per GPa. On the other hand, as shown in Figure 2d, the induced strain via the inverse piezoelectric effect is one order smaller than that by the pressure application (see also Note S5, Supporting Information). This means that the observed enhancement Δ*H* due to the electric field cannot be explained only by the modification of the PMA due to the reduction in the vdW gap.

To further discuss the mechanisms underlying the observed effects more quantitatively, we take into account the Dzyaloshinskii–Moriya interaction (DMI). The magnetic energy density *w*_e_ for the present system can be expressed as:

(2) $$w_{e}=-A{\left(\nabla m\right)}^{2}-K_{\mathrm{ani}}m_{z}^{2}+Dm\cdot \left(\nabla \times m\right),$$

where *A* represents the exchange stiffness constant, *K*_ani_ is the magnetic anisotropy constant, and *D* denotes the DMI constant. With regard to the ferromagnetic exchange interaction in Fe_3_GaTe_2_, our observations reveal that the Curie temperature *T*_C_ remains unchanged up to *E*_[001]_ = 6 kV/cm as indicated in Figure 3e. Additionally, as shown in Figure 5a, the saturation field (*H*_s_) displays minimal sensitivity to the applied electric field. Furthermore, the in‐plane strain in the PMN‐PT substrate induced by the electric field is less than 0.03%. These results strongly support the hypothesis that the ferromagnetic exchange stiffness constant (*A*) within the 2D layers of Fe_3_GaTe_2_ is not significantly influenced by the application of the electric field.

Although the strain induced by the electric field is relatively small, it permeates the entire thickness of the Fe_3_GaTe_2_ layer. The vdW gap is notably sensitive to strain, which modulates the perpendicular magnetic anisotropy. However, as previously discussed, these strain‐induced effects alone cannot fully account for the significant reduction in the nucleation field *H*_*n*_ under the electric field application. Importantly, the strain decreases progressively with distance from the interface, creating a strain gradient that suggests the relevance of the DMI. An enhancement in DMI can lead to the formation of non‐collinear spin structures, such as skyrmions, in ferromagnetic materials. Notably, room‐temperature skyrmions attributed to DMI have been reported in Fe_3_GaTe_2_.^[^ ^22^, ^23^, ^24^, ^25^ ^]^ DMI arises from the interplay of spatial inversion symmetry breaking and strong spin‐orbit coupling. In the Fe_3_GaTe_2_/PMN‐PT heterostructure studied here, strain gradients due to strain relaxation significantly break symmetry along the out‐of‐plane direction. Additionally, the anisotropic strain introduced by the single‐crystal PMN‐PT substrate, characterized by its fourfold symmetry, disrupts the intrinsic hexagonal symmetry of Fe_3_GaTe_2_. These combined factors contribute to the enhancement of DMI in Fe_3_GaTe_2_, as illustrated in Figure 5e.

To further investigate these phenomena, direct observations of magnetic domain behavior under electric field modulation would provide valuable insights. The ability to fabricate few‐atomic‐layer Fe_3_GaTe_2_ films directly on PMN‐PT with clean interfaces would allow for maximized strain coupling and could unveil more pronounced or novel electric‐field effects intrinsic to the ultrathin regime. The interface multiferroic structure formed by Fe_3_GaTe_2_ on PMN‐PT demonstrates the potential for controlling magnetic domain states with minimal electric power consumption. This capability offers promising applications in electric‐field‐driven micro‐electromechanical systems (MEMS) and next‐generation memory devices.

## 3 Conclusion

We experimentally investigated the electric‐field effects on the magnetic properties of a heterostructure composed of the vdW ferromagnet Fe_3_GaTe_2_ and the ferroelectric PMN‐PT. To evaluate the magnetic properties, AHE measurements were performed under varying back‐gate electric fields and temperatures. Application of an electric field resulted in a significant reduction of the domain nucleation field and markedly altered the magnetization reversal mechanismfrom coherent rotation in a single‐domain state to the formation of a multi‐domain structure. In contrast, no notable change in the Curie temperature, associated with ferromagnetic exchange interactions, was observed under electric field application. Our analysis revealed that the inverse piezoelectric effect induced by the PMN‐PT expanded the vdW gap, which not only weakened the interlayer perpendicular magnetic anisotropy but also enhanced the DMI in Fe_3_GaTe_2_ through symmetry reduction. These findings highlight the strong magneto‐elastic coupling properties of vdW ferromagnets and demonstrate the potential for electric‐field‐driven control of magnetic structures with minimal power consumption. This approach paves the way for advanced applications in next‐generation spintronic devices.

## 4 Experimental Section

### Device Fabrication and Characterization

In the present study, we fabricated the Fe_3_GaTe_2_/PMN‐PT heterostructure device by mechanically exfoliating single crystals consisting of Fe_3_GaTe_2_. The bulk Fe_3_GaTe_2_ crystals were synthesized using a self‐flux method. A mixture with a molar ratio of Fe:Ga:Te = 1:1:2 was placed in an alumina crucible, vacuum‐sealed in a quartz tube, and heat‐treated in a muffle furnace. The temperature was ramped to 1000 °C within 1 hour, maintained for 24 hours, then lowered to 880 °C within 1 hour, and gradually cooled to 780 °C over 120 hours. The temperature was held at 780 °C for 24 hours before centrifugation was employed to separate the crystals from the Te flux. Figure S1 (Supporting Information) presents the X‐ray diffraction (XRD) spectra of the synthesized Fe_3_GaTe_2_, which predominantly exhibit (00*h*) peaks, indicating a high‐quality crystal with *c*‐axis orientation. In addition, the transmission electron microscopy (TEM) image clearly reveals the layered crystal structure as shown in Figure 1b. Figure 1c illustrates the Hall measurement device structure. The Fe_3_GaTe_2_ film was mechanically exfoliated and transferred onto a (001)‐oriented single‐crystalline PMN‐PT substrate in a nitrogen‐filled glovebox. The thickness of the exfoliated Fe_3_GaTe_2_ film was measured to be 49 nm using atomic force microscopy (AFM), as shown in the right panel of Figure 3b. Subsequently, electrodes were patterned using electron beam lithography and low‐energy ion milling techniques. Figure 3a shows a scanning electron microscopy (SEM) image of the fabricated Fe_3_GaTe_2_/PMN‐PT device. Copper (200 nm) electrodes were deposited via Joule heating evaporation under a base pressure of 2.0 × 10^−6^ Pa. For back‐gating control, silver paste was applied to the back side of the PMN‐PT substrate to fabricate gate electrodes. To prevent property changes due to redox reactions, the fabricated devices were spin‐coated with poly(methyl methacrylate) (PMMA).

### Electric Field Control Using Ferroelectric PMN‐PT Single Crystals

The electric field control was performed using the programmable DC voltage source Advantest R6161. The electric field was symmetrically driven in a bipolar manner, alternating sequentially between |*E*_max_| and −|*E*_max_|, to prevent the retention of biased hysteresis. Furthermore, to prevent cracks caused by inhomogeneous strain, the uniform application of the gate electric field was verified using an optical microscope by confirming that the transparency changed uniformly with the application of the electric field. The detailed electric‐field‐induced strain characteristics of the PMN‐PT were evaluated using the XRD as shown in the inset of Figure 2b. As the strain‐induced peak shift becomes more pronounced at higher diffraction angles, we consequently selected the (003) peak at the highest accessible angle for our system. In the measurement of electric‐field‐induced peak shifts, it was necessary to evaluate the XRD spectra using the highest resolution available with our setup. To ensure efficient measurements and to avoid time‐dependent drift, the XRD scans were limited to the region around the (003) peak as shown in Note S2 (Supporting Information). Figure 2d shows the gate field *E*_[001]_ dependence of the out‐of‐plane strain ϵ_*zz*_ (=Δ*c*/*c*) for the PMN‐PT substrate. The strain curve was evaluated by the Bragg‐peak shift of the PMN‐PT (003) reflection. We observed the ferroelectric hysteresis and the tensile strain of about 0.05% along the *c*‐axis, which is consistent with the previous studies.^[^ ^45^ ^]^ Considering the constant volume Poisson effect (2ϵ_*xx*_ + ϵ_*zz*_ = 0), the *c*‐axis tensile strain corresponds to an in‐plane compressive strain ϵ_*xx*_ of about −0.03~%, suggesting that Fe_3_GaTe_2_ undergoes in‐plane compressive strain from the PMN‐PT interface as shown in Figures 1c and 2d.

### Magneto‐Electric Transport Properties Measurements

The anomalous Hall effect (AHE) measurements were performed using an AC lock‐in detection technique under temperature control from 300 to 400 K. The applied current was 100 µA at a frequency of 173 Hz. Here, the time constant of the lock‐in amplifier is 100 ms and the sweep rate of the external magnetic field is 25 Oe/s. All measurements were conducted in vacuum to prevent degradation of the device during experiments. For high‐temperature application from 300 to 400 K, a ceramic heater (MC2525H) manufactured by Sakaguchi electric heaters Co., Ltd. was used, and temperature control was achieved using a DC‐type temperature controller with a K‐type thermocouple.

### First‐Principles Calculations

To evaluate the effect of in‐plane substrate strain on Fe_3_GaTe_2_, first‐principles calculations based on density functional theory (DFT) were performed using the QUANTUM ESPRESSO package.^[^ ^46^, ^47^ ^]^ We employed pseudopotentials within the projector‐augmented‐wave (PAW) framework, using the Perdew–Burke–Ernzerhof (PBE) generalized gradient approximation (GGA) for the exchange‐correlation functional. The plane wave cutoff energy was set to 70 Ry, with a 12 × 12 × 4 *k*‐point mesh for self‐consistent calculations in the Brillouin zone. To account for van der Waals interactions, we applied Grimme's DFT‐D3 dispersion correction.^[^ ^48^, ^49^ ^]^ Based on the above, the lattice relaxation of bulk Fe_3_GaTe_2_ was performed under various in‐plane strain conditions to evaluate the change in the lattice parameter along the *c*‐axis. The detailed results are provided in Note S3 (Supporting Information).

## Conflict of Interest

The authors declare no conflict of interest.

## Author Contributions

R.I., H.S., and T.K. conceptualized and planned the project. T.K. supervised the project. R.I. fabricated samples. Y.K. evaluated the X‐ray diffraction measurements with support from R.I. R.I. and Y.K. performed transport measurements. Analyses of the data were done by R.I., Y.K., H.S., and T.K. R.I. wrote the manuscript with support from K.Y, H.S., and T.K. All authors discussed the results and commented on the manuscript.

## Supporting information

Supporting Information

## Data Availability

The data that support the findings of this study are available from the corresponding author upon reasonable request.
