# Supplementary material for: Electrical Manipulation of Magnetic Domain Structure in van der Waals Ferromagnetic Fe3GaTe2 Using Ferroelectric PMN‐PT Single Crystal
Source: Adv Sci (Weinh). 2025 Aug 27;12(39):e03530. doi: 10.1002/advs.202503530 (PMC12533304; doi:10.1002/advs.202503530)
Supplement: Supplementary file 1 — Supporting Information [file ADVS-12-e03530-s001.pdf]

ADVANCED SCIENCE
Open Access

Supporting Information

for *Adv. Sci.*, DOI 10.1002/advs.202503530

Electrical Manipulation of Magnetic Domain Structure in van der Waals Ferromagnetic Fe_3GaTe_2 Using Ferroelectric PMN-PT Single Crystal

*Riku Iimori, Yuta Kodani, Shaojie Hu and Takashi Kimura**

Supporting Information:
Electrical Manipulation of Magnetic Domain Structure in van der Waals Ferromagnetic Fe_3GaTe_2 Using Ferroelectric PMN-PT Single Crystal

Riku Iimori,[1] Yuta Kodani,[1] Shaojie Hu,[1,2] and Takashi Kimura[1,3,*]

[1]*Department of Physics, Kyushu University, 744 Motooka, Fukuoka, 819-0395, Japan*

[2]*College of Integrated Circuits and Optoelectronic Chips, Shenzhen Technology University, 3002 Lantian Road, Pingshan District, Shenzhen Guangdong, China, 518118*

[3]*Research Center for Semiconductor and Device Ecosystem, Kyushu University, 6-1 Kasugakoen, Kasuga, 816-8580, Japan.*

[*] Corresponding author: t-kimu@phys.kyushu-u.ac.jp

S1: FUNDAMENTAL PROPERTIES OF SYNTHESIZED Fe_3GaTe_2 CRYSTAL

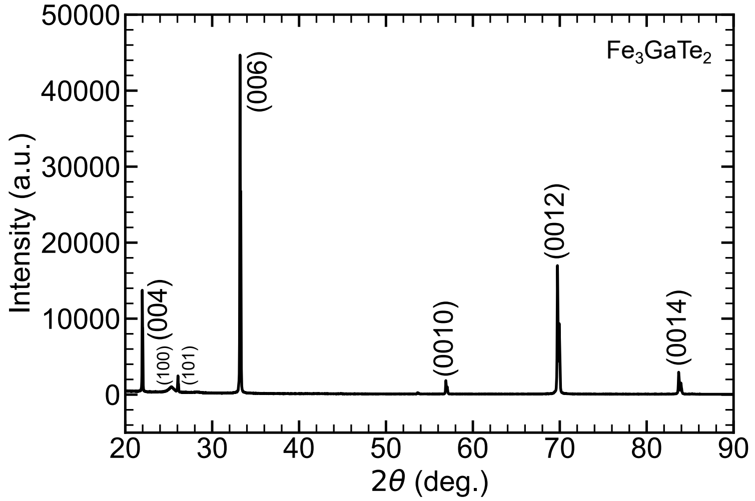

Supplementary Fig. S1: X-ray diffraction (XRD) pattern spectra of the synthesized bulk Fe_3GaTe_2 crystal.

Supplementary Figure S1 shows the X-ray diffraction (XRD) spectrum of the Fe_3GaTe_2 crystal plate. As described in the main text, the Bragg peaks corresponding to planes parallel to the *c*-plane were observed. In addition, we performed compositional analysis using energy-dispersive X-ray spectroscopy (EDX) as shown in Table I. As a result, we confirmed that stoichiometric crystals were successfully obtained in agreement with previous studies [S1, S2].

**Element**	**Weight%**	**Atomic%**
Fe	37.67 ± 4.29	53.81
Ga	13.92 ± 1.74	15.93
Te	48.41 ± 3.70	30.26
**Totals**	**100.00**	**100.00**

TABLE I. Elemental composition of the Fe_3GaTe_2 crystal.

S2: BRAGG PEAK SHIFT OF PMN-PT UNDER AN ELECTRIC FIELD

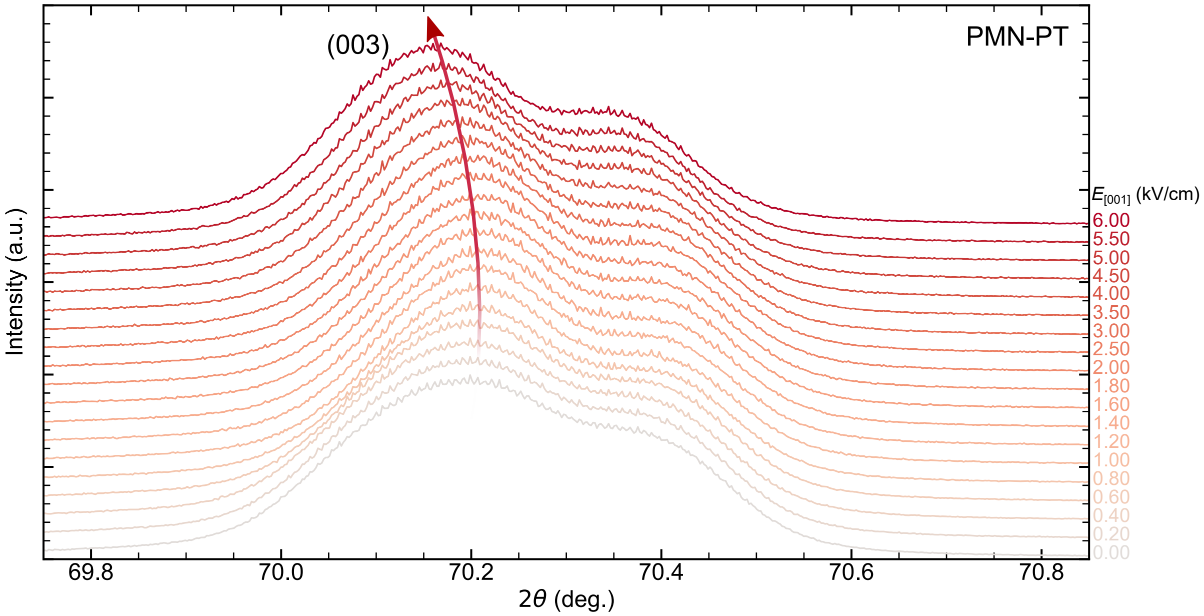

Supplementary Fig. S2: Electric-field-induced shift of the (003) Bragg peaks in PMN-PT. The electric field is applied along the [001] direction of PMN-PT. The strain curve of PMN-PT shown in Fig. 2(d) of the main text was evaluated based on these data.

S3: CALCULATION OF STRAIN PROFILE IN Fe_3GaTe_2

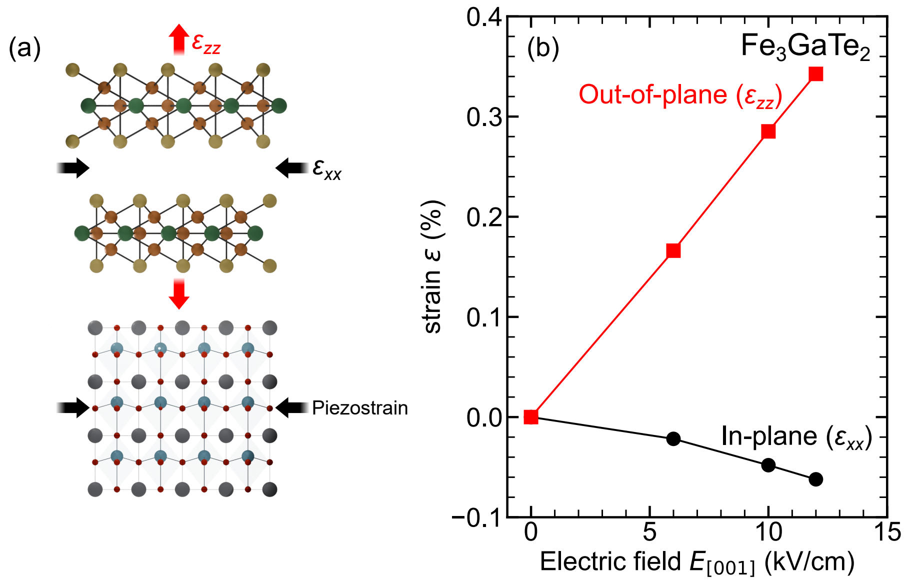

Supplementary Fig. S3: (a) Schematic diagram of the strain propagation due to piezostrain in the Fe_3GaTe_2/PMN-PT heterostructure. (b) Electric field $E_{[001]}$ dependence of the strain in the Fe_3GaTe_2.

Based on first-principles calculations, the strain characteristics of the Fe_3GaTe_2 on a ferroelectric PMN-PT single crystal were evaluated. As shown in Supplementary Fig. S3(a), the piezostrain of PMN-PT propagates to Fe_3GaTe_2 in the Fe_3GaTe_2/PMN-PT heterostructure. As a result, compressive strain ε_{xx} is applied in the in-plane direction, while tensile strain ε_{zz} is applied in the out-of-plane direction to the Fe_3GaTe_2 layer. Supplementary Figure S3(b) shows the gate electric field $E_{[001]}$ dependence of various strains in Fe_3GaTe_2. In the calculations, the in-plane strain corresponding to the electric field $E_{[001]}$ was applied, and the lattice parameter c and atomic positions were relaxed. The relationship between the electric field $E_{[001]}$ and the in-plane strain ε_{xx} was determined using the XRD results of PMN-PT under an electric field, and values above 10 kV/cm were obtained by linear extrapolation. As a result, the in-plane compressive strain induced a substantial expansion along the c-axis direction. This significant expansion along the c-axis direction is attributed to the enlargement of the van der Waals (vdW) gap. In fact, the vdW gap experiences approximately 1 % tensile strain.

S4: CYCLE DEPENDENCE OF ELECTRIC FIELD EFFECT: THE ROBUST DEVICE PROPERTIES

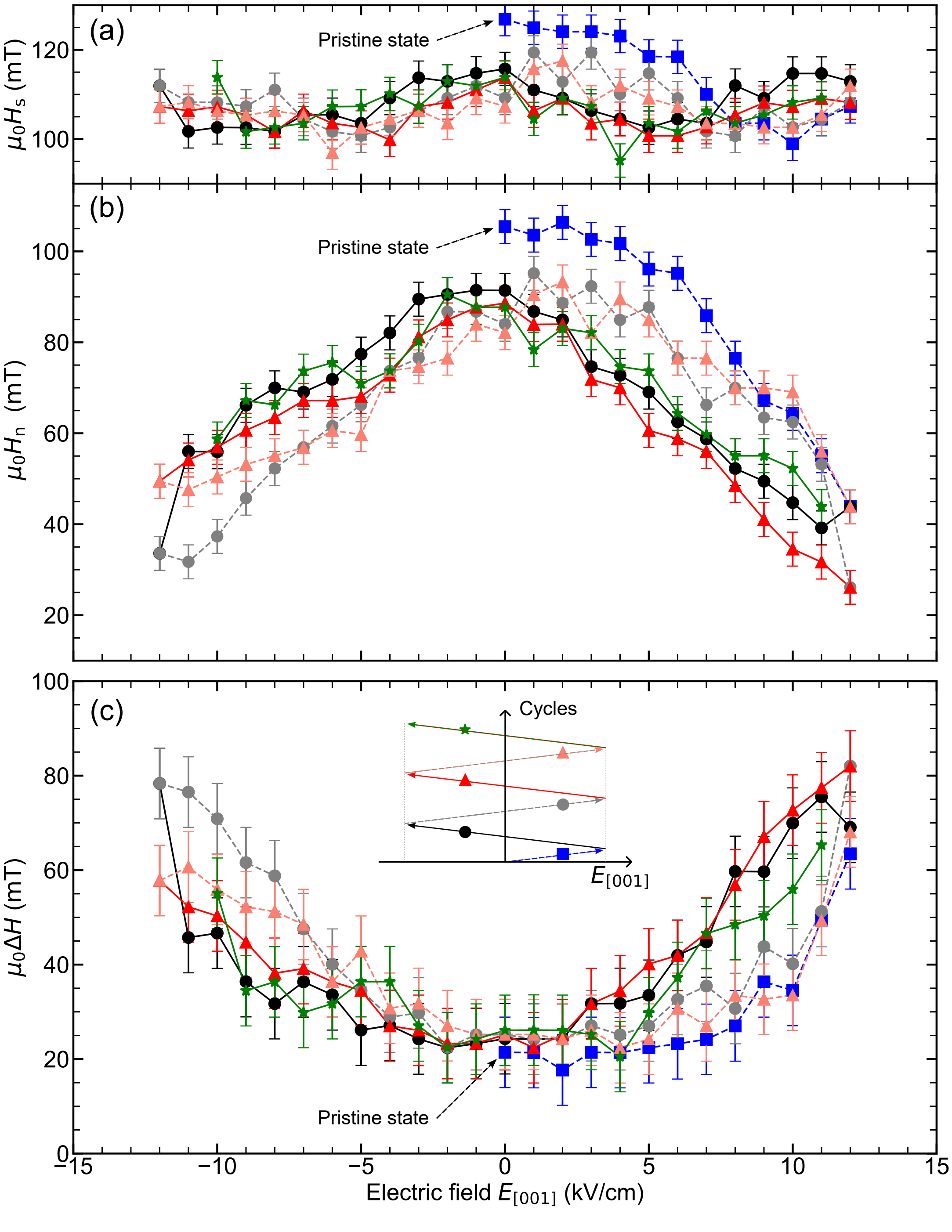

Supplementary Fig. S4: Cycle dependence of electric field effect on (a) the saturation magnetic field H_s, the nucleation magnetic field H_n and $\Delta H (= H_s - H_n)$. After the pristine state, the electric field effect appears reproducibly.

We also evaluated the reproducibility of the electric field effect on the magnetization process in the Fe_3GaTe_2/PMN-PT device as shown in Supplementary Fig. S4. Significant changes are observed in the initial pristine state, likely due to strain relaxation introduced during device fabrication. In contrast, the subsequent electric field cycles exhibit consistent and reproducible variations in H_s, H_n and ΔH, clearly demonstrating the robustness of the device. Moreover, the butterfly-like shape consistently observed in all sweep cycles confirms that the electric-field-induced changes arise from strain effects imposed by the PMN-PT substrate.

S5: COMPARISON OF PIEZOSTRAIN AND HYDROSTATIC PRESSURE: FOCUSING ON CHANGES IN THE VAN DER WAALS GAP

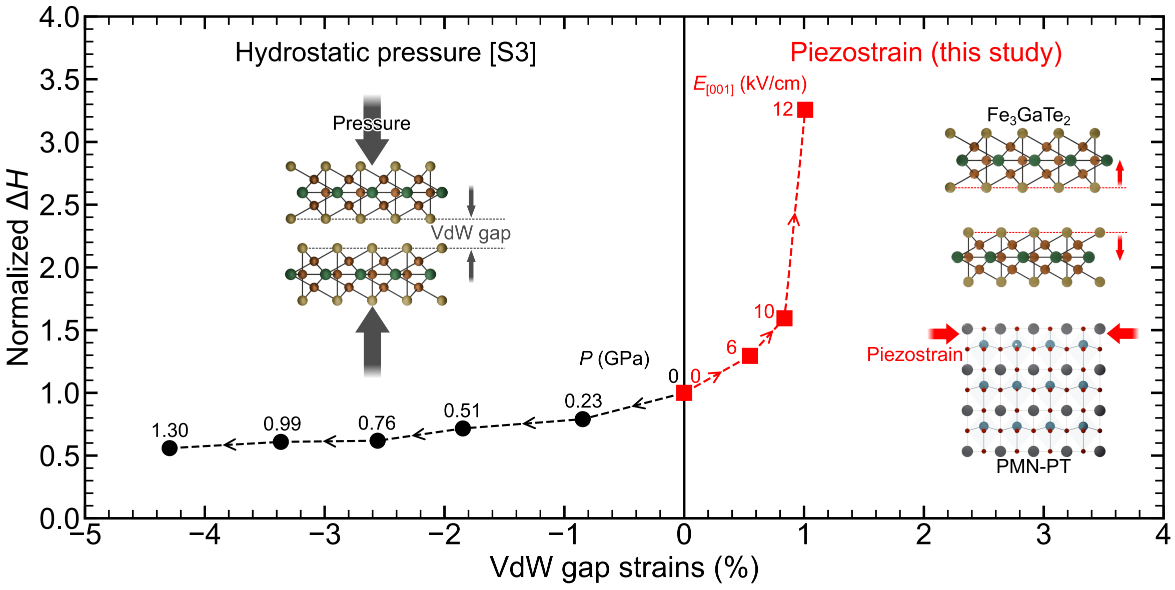

Supplementary Fig. S5: Comparison of ferroelectric piezostrain (this work) and hydrostatic pressure experiments (our previous work [S3]). Normalized plot of ΔH as a function of the van der Waals (vdW) gap, where ΔH represents the difference between the saturation magnetic field H_s and the nucleation magnetic field H_n.

Finally, we compared the results of this study with our previous hydrostatic pressure experiments [S3]. In contrast to the piezostrain study in this work, the vdW gap decreases in the hydrostatic pressure experiments as shown in Supplementary Fig. S5. In the hydrostatic pressure experiments, the reduction of the vdW gap enhanced the interlayer magnetic coupling, leading to an improvement in perpendicular magnetic anisotropy. As a result, ΔH decreased, suggesting a suppression of the multi-domain state. In contrast, the piezostrain applied in this study results in an expansion of the vdW gap, weakening the perpendicular magnetic anisotropy. However, the piezostrain causes a more pronounced change in ΔH compared to hydrostatic pressure. This difference is considered to be due to the enhancement of the Dzyaloshinskii-Moriya interaction.

[S1] Gaojie Zhang, Fei Guo, Hao Wu, Xiaokun Wen, Li Yang, Wen Jin, Wenfeng Zhang, and Haixin Chang. Above-room-temperature strong intrinsic ferromagnetism in 2D van der Waals Fe_3GaTe_2 with large perpendicular magnetic anisotropy. *Nat Commun* **13**, 5067 (2022).

[S2] Shuhan Liu, Shaojie Hu, Xiaomin Cui, and Takashi Kimura. Efficient Thermo-Spin Conversion in van der Waals Ferromagnet FeGaTe. *Adv. Mater.* **36**, 2309776 (2024).

[S3] Riku Iimori, Shaojie Hu, Akihiro Mitsuda, and Takashi Kimura. Substantial enhancement of perpendicular magnetic anisotropy in van der Waals ferromagnetic Fe_3GaTe_2 film due to pressure application. *Commun Mater* **5**, 235 (2024).
